# Excessive Daytime Sleepiness and REM Sleep Behavior Disorders in Parkinson's Disease: A Narrative Review on Early Intervention With Implications to Neuroprotection

**DOI:** 10.3389/fneur.2018.00961

**Published:** 2018-11-14

**Authors:** Michaela D. Gjerstad, Guido Alves, Jodi Maple-Grødem

**Affiliations:** ^1^The Norwegian Centre for Movement Disorders, Stavanger University Hospital, Stavanger, Norway; ^2^Department of Chemistry, Bioscience and Environmental Engineering, University of Stavanger, Stavanger, Norway; ^3^Department of Neurology, Stavanger University Hospital, Stavanger, Norway

**Keywords:** early Parkinson's disease, sleep, genetics, EDS, RBD

## Abstract

Sleep contributes to the consolidation of our memory and facilitates learning. Short term sleep deprivation temporarily reduces mnestic capacity, whereas long lasting sleep deprivation is associated with structural changes in the hippocampus and cortical areas. However, it is unknown whether early intervention and treatment of sleep disorders could have a neuroprotective effect. In neurodegenerative diseases sleep disorders can occur at preclinical stages and are frequently observed in patients with established Parkinson's disease (PD) and other α-synucleinopathies. REM sleep behavior disorder (RBD) is recognized as a hallmark for the development of α-synucleinopathies and may predict early cognitive decline, while excessive daytime sleepiness (EDS) is present in 12% of patients with PD before treatment initiation and increases continuously over time, causing substantial restrictions for the patients' social life. In more advanced disease, EDS is associated with dementia. Even though well recognized, limited attention has been given to genetics or the treatment of RBD and EDS in early PD. Systematic screening and early intervention can be expected to increase the patients' quality of life, but it remains unclear if this will also impact disease progression. Intervention studies in preclinical and early stages of α-synucleinopathies are needed to increase our understanding of the underlying pathomechanisms and may also provide important inroads to help clarify whether sleep disturbances are secondary to the neurodegenerative process or also contribute to disease exacerbation.

## Introduction

The wish for restorative sleep is equal for any human. Close to one third of the human life is spent asleep and perfect sleep means awakening refreshed and oblivious to the hours spent at rest. A number of conditions have to be satisfied in order to sleep well. These include the duration, continuity, rhythmicity, quality, and amount of time spent in different sleep stages. Short term sleep disruption can temporarily impede most behavioral processes (1) and lead to deficits in attention, executive function, non-declarative and declarative memory, and emotional reactivity and sensory perception (2, 3). Conversely, a good night's sleep or daytime napping has significant beneficial effects on human alertness and experienced well-being (4, 5), and the synaptic homeostasis hypothesis indicates sleep as the essential phase to facilitate plasticity of the brain and memory consolidation, by reducing the burden of plasticity on neurons while restoring neuronal selectivity and the ability to learn (6). The complexity of our sleep/wake system makes sleep vulnerable to disturbances, which then can lead to adverse health outcomes.

Sleep quality and duration change with aging (7). The increasingly disturbed sleep in the elderly is caused by a weakening of the circadian system with a blunted diurnal melatonin level (8), as well as, changes in the sleep and wake regulating systems (9). Reciprocally, weakened sleep structures render the patient prone to the development of sleep disturbances and subsequently reduced daytime function. Other reasons for sleep disorders in the elderly may be the occurrence of diseases causing sleep disruption or medication.

It remains unclear to what extent disturbances of sleep caused by aging or disease contribute to the development of neurodegenerative diseases or whether they are only a byproduct of other conditions. Further, it is unknown if early intervention to treat sleep disturbances may delay the neurodegenerative processes. This viewpoint will address these matters, focusing on two major sleep disorders: Excessive daytime sleepiness (EDS) and REM sleep behavior disorder (RBD). EDS and RBD occur frequently in early α-synucleinopathies and have possible implications for further disease development. Early detection and intervention may clarify parts of the riddle as to what extent sleep disturbances contribute to the cause or the consequence of PD. Finally, sleep deprivation will be shortly discussed as increasing evidence indicates that this may also be a key player in these neurodegenerative diseases.

## Early parkinson's disease and sleep disorders

Disturbed or impaired sleep is very common in PD. The most frequent sleep disturbances recorded in PD are EDS, RBD and insomnia. The pathogenesis of these sleep disturbances may be attributed to the underlying disease pathologies involving the brainstem and the hypothalamus, or be the consequence of indirect mechanisms, for example dopaminergic medication or PD-related motor and non-motor symptoms (10–12). EDS is typically more prevalent in later stages of the disease, whilst RBD may be present years before the classic clinical features of PD and other α-synucleinopathies such as dementia with Lewy bodies occur (13).

Several subtypes of PD exist: While age of onset (14) and motor-subtype (15) have long been recognized as important prognostic factors for the course of PD, certain non-motor patterns are now also considered to indicate different neuropathological pathways and progression rates (16). Sleep phenotypes observed in PD are proposed to be associated with specific patterns of pathological progression, which can have subsequent impact on clinical presentations. For example, the brainstem-dominant phenotype is reported to relate to non-motor symptoms, such as olfactory disturbances and psychiatric symptoms (17). These findings remain debated but may be useful to indicate prognosis or treatment response, and more studies of the natural history of sleep disturbances and disease progression from premotor- and early motor-stages of disease will help to clarify if sleep symptoms help to define specific PD subtypes.

## RBD in early PD

RBD is a parasomnia triggered by lower brainstem dysfunction, resulting in lack of physiological atonia during REM sleep (18). Consequently, patients may enact their dreams physically or vocally during REM sleep (19). RBD is a major risk factor for the development of α-synucleinopathies, occurring in 20–75% of patients (20, 21). It has been associated with more severe motor symptoms and signs, and more depression/anxiety (16), and was found to be one of the strongest clinical predictors of cognitive impairment after older age (22).

RBD is accepted as a risk marker and in the context of research is increasingly used to estimate likelihood ratios of developing PD. Patients with incident RBD without Parkinsonism will on average be diagnosed with an α-synucleinopathy within a decade of the onset of the sleep disorder (23). The emergence of RBD can be attributed to the initial α-synuclein pathology associated dysfunction in the brainstem, which later ascends to more rostral structures. The motor symptoms only manifest after the loss of about 80% of the substantia nigra cells (24, 25). Furthermore, a PET study detected microglial activation in the substantia nigra of patients with idiopathic RBD (26), implicating neuroinflammation in the early stages of α-synucleinopathies.

Not all patients with PD will display clinically relevant RBD, indicating that additional factors are involved in the co-occurrence of RBD and PD. Several genetic variants have been identified, which in addition to influencing susceptibility for PD, may also affect the risk of developing RBD. Among these are pathogenic variants in *GBA*, the gene encoding for the enzyme Glucocerebrosidase, which are associated with idiopathic RBD and worsening of the frequency of symptoms of RBD over time in both the non-PD population (27, 28) and in PD patients (27, 29–31). RBD also preceded the onset of the cardinal motor symptoms of PD SNCA p.A53T carriers (32), although the association of RBD with more common PD-risk *SNCA* variants is inconclusive (33–35). In contrast, polymorphism in *SCARB2* and *MAPT* have been associated with a reduced risk of developing PD or RBD in the non-PD population (33, 36). Combining genetic and clinical sleep data may assist in identifying individuals susceptible to PD in the prodromal phase, and in doing so offer an important window of time for neuroprotective treatment or lifestyle interventions.

## EDS in early PD

EDS in PD is defined as an inappropriate increased sleep propensity or increased need of time spent asleep, and is most frequently measured with the Epworth Sleepiness Scale (positive if score >10) (37). EDS causes frequent, major social problems and may interfere with the patients' driving abilities. The increase in frequency of EDS with disease duration and severity may be explained by an advancing neurodegenerative process. Other causes for EDS in PD are the potential sedating effect of dopaminergic medication, as well as secondary increased sleep propensity during daytime due to dysregulated and insufficient night time sleep (38).

Occurrence of EDS in the general population has been associated with the development of dementia and especially AD (39, 40). It remains uncertain to what extent EDS may precede the development of Parkinsonism and whether the occurrence of EDS in early PD foretells a certain disease progression. The only two studies to examine EDS longitudinally in the general population report a higher risk for the subsequent development of PD (41, 42). In our population based incident cohort study, drug naïve patients with PD reported more frequent EDS compared to age and sex matched controls, and an increased Epworth Sleepiness Scale score at baseline was found to be the main risk for the subsequent development of EDS (43). Nevertheless, results remain contrary, with findings of both increased and equivalent prevalence of EDS compared to matched healthy control subjects (44, 45). To avoid the confounding influence of dopaminergic treatment on sleepiness more longitudinal population studies are needed, examining drug naive patients and the role of EDS as a prodromal or associated feature in early PD.

There are no proven genetic risk factors for EDS in PD. Several studies have investigated a link between EDS and the Catechol O-methyltransferase (COMT) val158met polymorphism, which affects synaptic dopamine levels following neurotransmitter release, but the results are inconsistent (46, 47). Interestingly, in the Sleep Heart Health Study, daytime sleepiness was found to be associated with an intronic variant in the gene encoding phosphodiesterase 4D (PDE4D) (48). PDE4D is implicated in memory consolidation, one of the functions of sleep, and might represent a therapeutic target for cognition enhancement. Furthermore, in PD, *PDE4D* is significantly hypermethylated compared to controls (49), and PDE4 inhibitors have been shown to have a neuroprotective effect in mice treated with MPTP (50), whilst broad spectrum PDE inhibitors protect cultured neurons against amyloid-beta (Aβ) and α- synuclein-induced synapse damage (51).

## Lessons from other fields: the role of sleep deprivation

Mounting evidence points to short- or long-term sleep deprivation as a cause to structural and pathological changes in the brain. A number of animal studies document the sensitivity of the hippocampus to chronic sleep deprivation (52) and an imaging study reported increased neuronal loss in the hippocampus of patients with chronic insomnia (53). In mice, sleep deprivation promotes astrocytic phagocytosis and microglial activation (54), likely leading to exacerbated phagocytosis of synaptic elements.

Several studies also show that sleep is the most important diurnal phase for clearance of neuronal metabolites such as Aβ42 (55–57). In addition, the reduction of slow wave sleep increase the level of brain Aβ prior to amyloid deposition, the hallmark of Alzheimer's disease (AD), which is also observed in patients with dementia with Lewy bodies (DLB) and PD (58), indicating that interventions targeting sleep that reduce amyloid burden could be of significance in the prevention or treatment of both AD and α-synucleinopathies.

## Genetic influences on sleep: clues for intervention strategies

Healthy people vary in their preferences for sleep timing and length, and response to sleep deprivation and susceptibility to sleep disorders varies from person to person. Although environmental factors can account for much of this variability, an individual's underlying genetic architecture (including genetic mutations and polymorphisms, and epigenetic changes) undoubtedly influences sleep. As discussed, relatively little is known about the role of genetic variants in sleep-related disorders in α-synucleinopathies, but recent advances in the study of circadian genes and epigenetics in other fields suggests possible targets for intervention therapies.

Sleeping and waking outside of the times set by the internal circadian clock can cause negative health outcomes, including neurological issues. In the general population, mistimed sleep, like that associated with jet lag or shift work, disrupts the rhythms of hundreds of genes, including key regulators of gene expression and core clock genes, notably *CLOCK* and *ARNTL* (*BMAL1*) (59). Interestingly, the phase and amplitude of the clock genes may also be altered in PD (60–62), proposing that sleep dysfunction seen in early PD may reflect an underlying pathology in the molecular clock. The molecular mechanisms that disrupt circadian regulation in PD are not clear, however patients with α-synucleinopathies exhibit DNA methylation changes associated with clock genes, for example decreased methylation of the *NPAS2* gene promoter from PD patients (63) and of *PER1* and *CRY1* from DLB patients (64) leukocytes have been reported. Although effects on the central circadian clock have not been shown, aberrant DNA methylation of key clock genes in the PD brain may potentiate widespread circadian deregulation and the development of sleep disorders and/or PD. Alternative theories point to the role of progressive dopaminergic loss within the nigrostriatal system, since dopamine both regulates and is regulated by the clock genes in the hypothalamic suprachiasmatic nucleus and peripheral brain areas (65, 66).

The association between dopaminergic therapies and circadian genetic markers in PD has not been investigated, but animal models have demonstrated increased mRNA levels of selected clock genes after application of D_1_ and D_2_ dopamine receptor agonists (67), while D_2_ dopamine antagonists blunted the rhythm of striatal *PER2* (68). These observations have implications for circadian abnormalities seen in PD, especially in medicated patients or in advanced disease.

## Discussion

Sleep problems are among the earliest symptoms of PD and increase with disease progression. RBD and EDS are the most common documented disorders affecting the patient, whereas research on disruption of the circadian system is just at the beginnings. It is not yet known if early treatment of sleep disorders could reduce the risk of developing α-synucleinopathies or slow disease progression, but as our understanding of the restorative role that sleep plays increases (6), the suggestion that interventions targeting sleep disorders will have positive implications for α-synucleinopathy disease susceptibility and progression gains credence.

To date there is limited evidence that behavioral or pharmaceutical interventions to regularize sleep/wake activity might be therapeutically useful in neurodegenerative disease. As in humans, sleep deprivation in mice can cause degeneration of neurons. Mouse models of Huntington's disease (HD) display degeneration of sleep rhythms, and early pharmacological intervention to restore sleep by treatment with the sedative clonazepam at the onset of the light phase, normalizes clock gene oscillation in these mice and significantly improves cognitive performance in this model (69). More recently, the motor symptoms of the HD mice were also alleviated following early time-restricted feeding intervention that improved circadian rhythmicity (70). These observations give hope that treatments or lifestyle interventions aimed at restoring circadian rhythms are promising targets to slow the neurodegenerative processes and could also improve other circadian gene-regulated functions that are impaired in PD.

Sleep disturbances, especially insomnia, in young adults seem to have the potential to cause structural changes of the brain. To what extent this may have health consequences decades later one can only speculate. A recent published Nordic study reports a 1.24 and 1.94 hazards ratio for the risk of developing dementia 3–10 years following midlife and late-life insomnia, respectively (71). However, it remains uncertain whether insomnia is one of the causes or a consequence of neurodegenerative processes or a combination of both. Effects of sleep deprivation have mainly been shown to be associated to amyloid deposition, and there are an increasing number of reports referring to co-existing amyloidopathy in PD patients with dementia (72, 73). Whether these patients experienced more or serious insomnia is to our knowledge not yet shown, though investigating a possible association is of interest. In AD, how the amount and quality of sleep affects Aβ aggregation is not fully understood, but animal studies indicate the importance of delaying the onset of this pathology in impacting the time of onset of disease, and such manipulation may be a powerful way to modulate amyloid pathology in the preclinical stages of disease.

Sleep disturbances in young and middle-aged adults may herald the pathological processes leading to α-synucleinopathies. Considering the negative health implications of long-term sleep disruption, they may even potentiate the development of neurodegenerative disease. This might be augmented in some individuals with a genetic predisposition to circadian dysfunction or neurodegeneration, for example genetic variants in circadian clock genes are associated with susceptibility to PD and other neurodegenerative disorders (74–76), and to cognitive impairment in the general elderly population (77). Further genetic and epigenetic factors influencing the aging process, response to treatment, or susceptibility to sleep disorders directly, can also be expected to play a role in modulating sleep disorders and the underlying neurodegenerative processes, and will provide useful insights into the biological basis of these disorders, and potential targets for future intervention therapies.

To establish if and when early treatment of sleep disturbances changes the course of neurodegenerative disease, there is a need for longitudinal population studies of the natural history of the development of sleep disorders and the conversion to α-synucleinopathies. The focus should be on early changes in circadian rhythms, sleep deprivation, EDS and RBD several decades before the manifest occurrence of motor symptoms in PD. Such work will bring us closer to the goal of intervention studies, by revealing the severity and timing of the onset of circadian disturbances in sufferers of PD compared to the occurrence in the otherwise healthy aging population. The enrollment of patients with idiopathic RBD or carriers of mutations in *GBA* are promising strategies to enrich studies for individuals at risk of converting to PD. Collaborative efforts between cohorts will also hasten progress to understand the implications of maximizing sleep health on the prevalence of PD and α-synucleinopathies in the future generations.

## Author contributions

MG contributed conception of the review. MG and JM-G wrote the first draft of the manuscript. GA wrote sections, and critically reviewed the manuscript. All authors contributed to manuscript revision, read and approved the submitted version.

### Conflict of interest statement

The authors declare that the research was conducted in the absence of any commercial or financial relationships that could be construed as a potential conflict of interest.
